# Analysis of intracellular expressed proteins of *Mycobacterium tuberculosis *clinical isolates

**DOI:** 10.1186/1477-5956-10-14

**Published:** 2012-03-01

**Authors:** Neelja Singhal, Prashant Sharma, Manish Kumar, Beenu Joshi, Deepa Bisht

**Affiliations:** 1 Department of Biochemistry, National JALMA Institute for Leprosy and other Mycobacterial Diseases, Tajganj, Agra 282001, India; 2 Department of Biophysics, University of Delhi, South Campus, Benito Juarez Road, New Delhi 110021, India; 3 Department of Immunology, National JALMA Institute for Leprosy and other Mycobacterial Diseases, Tajganj, Agra 282001, India

## Abstract

**Background:**

Tuberculosis (TB) is the most threatening infectious disease globally. Although progress has been made to reduce global incidence of TB, emergence of multidrug resistant (MDR) TB threatens to undermine these advances. To combat the disease, novel intervention strategies effective against drug resistant and sensitive subpopulations of *M. tuberculosis *are urgently required as adducts in the present treatment regimen. Using THP-1 cells we have analyzed and compared the global protein expression profile of broth-cultured and intraphagosomally grown drug resistant and sensitive *M.tuberculosis *clinical isolates.

**Results:**

On comparing the two dimensional (2-DE) gels, many proteins were found to be upregulated/expressed during intracellular state which were identified by matrix assisted laser desorption/ionization mass spectrometry (MALDI-MS). Four proteins (adenosylhomocysteinase, aspartate carbomyltransferase, putatitive thiosulfate sulfurtransferase and universal stress protein) were present in both intracellular MDR and sensitive isolates and three of these belonged to intermediary metabolism and respiration category. Two proteins (alanine dehydrogenase and adenosine kinase) of intracellular MDR isolate and two (glucose-6-phosphate isomerase and ATP synthase epsilon chain) of intracellular sensitive isolate belonged to intermediary metabolism and respiration category. One protein (Peroxidase/Catalase) of intracellular MDR and three (HSPX, 14 kDa antigen and 10 kDa chaperonin) of sensitive isolate belonged to virulence, detoxification and adaptation category. ESAT-6 of intracellular MDR belonged to cell wall and cell processes category. Two proteins (Antigen 85-C and Antigen 85-A) of intracellular sensitive isolate were involved in lipid metabolism while probable peptidyl-prolyl cis-trans isomerase A was involved in information pathways. Four (Rv0635, Rv1827, Rv0036c and Rv2032) of intracellular MDR and two proteins (Rv2896c and Rv2558c) of sensitive isolate were hypothetical proteins which were functionally characterized using bioinformatic tools. Bioinformatic findings revealed that the proteins encoded by Rv0036, Rv2032c, Rv0635, Rv1827 and Rv2896c genes are involved in cellular metabolism and help in intracellular survival.

**Conclusions:**

Mass spectrometry and bioinformatic analysis of both MDR and sensitive isolates of *M. tuberculosis *during intraphagosomal growth showed that majority of commonly upregulated/expressed proteins belonged to the cellular metabolism and respiration category. Inhibitors of the metabolic enzymes/intermediate can therefore serve as suitable drug targets against drug-resistant and sensitive subpopulations of *M. tuberculosis*.

## Background

Despite more than a century of research, tuberculosis (TB) as a disease claims more deaths than any other infectious agent making its causative organism *Mycobacterium tuberculosis*, one of the most successful human pathogens. Inappropriate treatment regimens and patient poor-compliance have led to the appearance of drug resistant TB. Multi Drug Resistant TB (MDR-TB) is caused by bacteria that are resistant to the most effective anti-TB drugs (Isoniazid and Rifampicin) with or without resistance to other drugs. 50% of MDR-TB cases in world are estimated in India and China [1]. In 2010, the largest WHO MDR-TB survey reported the highest rates of MDR-TB, with 28% of new TB cases in some settings of the former Soviet Union [1]. This is an alarming situation which calls for exploring therapeutics equally effective against drug sensitive and resistant population of *M.tuberculosis*. A major impetus of TB drug development process is to develop chemical compounds capable to cure TB patients, regardless of whether the disease is caused by *M.tuberculosis *which is drug sensitive or resistant to the current first and second line drugs [2]. Identification and development of improved intervention strategies requires better understanding of host-pathogen interactions. Different approaches have been used to study mycobacterial genes that play a role in the interaction with host cells and thus in virulence. These include *in vivo*- induced antigen technology, subtractive hybridization, *in vivo *expression technology etc. [3-5].

Proteomics is a powerful tool to study complex biological samples and its application has greatly contributed to a better understanding of the biology of *M.tuberculosis *and other pathogenic bacteria. Putative drug targets, vaccine candidates, and diagnostic markers for TB have also been identified using this approach [6-8]. Identification of mycobacterial proteins of drug resistant and sensitive isolates by two-dimensional electrophoresis (2-DE) and mass spectrometry has largely been applied to broth grown cultures, because abundant amounts of protein are available here for analysis and comparison. Such studies have been carried out by us [9] and other researchers [10,11]. Difficulty in recovery of sufficient amounts of protein from intracellular state accounts for the existence of only a few such studies [12,13]. To date, our knowledge regarding the proteomic profiles of drug resistant and sensitive *M.tuberculosis *during intracellular growth have been fragmentary.

In the present work, we have analyzed the protein expression profile of *M.tuberculosis *MDR and sensitive isolates while infecting THP-1 cells to study the gene expression changes that actually affect survival and growth of resistant or sensitive isolates while growing inside the host macrophage cells to identify proteins or protein-class which are upregulated/expressed inside macrophages and could be used as a common drug target for both types of microbial population.

## Results

**Figure 1:**
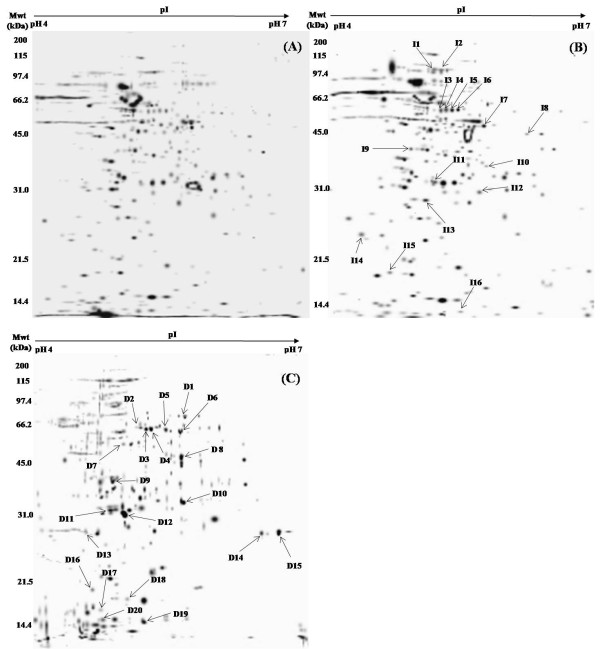
**Coomassie stained composite 2-DE gels of *M. tuberculosis *clinical isolates, cell lysate proteins run on 17 cm IPG strip of pH 4-7 and SDS-PAGE was performed on 22 cm × 20 cm gel**. (A) broth-cultured *M.tuberculosis *MDR and sensitive clinical isolates (B) intraphagosomally grown *M.tuberculosis *MDR clinical isolate and (C) intraphagosomally grown *M.tuberculosis *drug sensitive clinical isolate.

The goal of this study was to identify mycobacterial proteins upregulated/expressed during growth in macrophages by comparative proteome analysis of broth-cultured and intraphagosomally grown mycobacteria. To elucidate protein spots unique to intraphagosomal mycobacteria the generated 2-DE gels (Figure 1) were analyzed using the software program PDQuest (Biorad, USA) and spots which were upregulated/expressed with at least 2.5 intensity were selected.

### 2-DE of intracellular *M.tuberculosis *MDR isolate proteins and their MALDI-MS analysis

Comparison of composite 2-DE gels of broth-cultured *M. tuberculosis *MDR and sensitive isolates with intraphagosomally grown *M.tuberculosis *MDR isolate showed sixteen protein spots to be upregulated/expressed by the intraphagosomal mycobacteria (Figure 1B). Spots I1 and I2 (Rv1908c) were identified as peroxidase/catalase. Spots I3, I4, I5, I6 (Rv3248c) were identified as isoforms of Adenosylhomocysteinase. I7 (Rv2623) was identified as a Universal stress protein. Spots I8 (Rv2780c) and I9 (Rv2202c) were identified as Alanine dehydrogenase and Adenosine kinase respectively. Spots I10 (Rv2032), I13 (Rv0036c), I14 (Rv1827) and I15 (Rv0635) were identified as hypothetical proteins. Spot I12 (Rv1380) was identified as Aspartate carbamoyltransferase. Spot I11 (Rv0815c) was identified as Putative thiosulfate sulfurtransferase. Spot I16 (Rv2347c) was identified as a putative ESAT-6 like protein. Table 1 shows details of upregulated/expressed proteins identified by mass spectrometry.

**Table 1:** Details of over-expressed proteins of *M.tuberculosis *MDR isolate identified by mass spectrometry

**Spot No**.	Protein identified	MASCOT Score	Nominal Mass (Da)	pI	Sequence Coverage %	Accession Number
I1	Peroxidase/catalase T	144	80725	5.09	31%	Rv 1908c

I2	Peroxidase/catalase T	64	80725	5.09	9%	Rv 1908c

I3	Adenosylhomocysteinase (AdoHcyase)	105	54461	5.07	23%	Rv 3248c

I4	Adenosylhomocysteinase (AdoHcyase)	120	54461	5.07	25%	Rv 3248c

I5	Adenosylhomocysteinase (AdoHcyase)	88	54461	5.07	19%	Rv 3248c

I6	Adenosylhomocysteinase (AdoHcyase)	80	54461	5.07	17%	Rv 3248c

I7	Universal stress protein	162	31747	5.46	57%	Rv2623

I8	Alanine dehydrogenase	90	38974	5.81	31%	Rv2780c

I9	Adenosine kinase	108	34621	5.14	31%	Rv2202c

I10	Hypothetical protein	82	36765	5.28	23%	Rv2032

I11	Putative thiosulfate sulfurtransferase	37	31110	5.14	24%	Rv0815c

I12	Aspartate carbamoyltransferase (ATCase)	50	33798	6.60	22%	Rv1380

I13	Hypothetical protein	95	27586	4.84	44%	Rv0036c

I14	Hypothetical protein	64	17240	4.29	34%	Rv1827

I15	Hypothetical protein	85	17584	4.51	51%	Rv0635

I16	Putative ESAT-6 like protein	57	10970	5.17	29%	Rv2347c

### 2-DE of intracellular *M.tuberculosis *drug sensitive isolate proteins and their MALDI-MS analysis

Comparison of composite 2-DE gels of broth-cultured *M. tuberculosis *MDR and sensitive isolates with intraphagosomally grown *M.tuberculosis *sensitive isolate showed twenty one protein spots which were upregulated/expressed by the intraphagosomal mycobacteria (Figure 1C). Spot D1 (Rv0946c) was identified as Glucose-6-phosphate isomerase. Spots D2, D3, D4, D5, D6, (Rv3248c) were identified as isoforms of Adenosylhomocysteinase. Spots D7 (Rv2896c) and D13 (Rv2558) were identified as a hypothetical proteins. D8 (Rv2623) was identified as a Universal stress protein. Spots D9 (Rv0129c) and D12 (Rv3804c) were identified as Antigen 85-C and Antigen 85-A mycolyl transferases. Spot D10 (Rv1380) was identified as Aspartate carbamoyltransferase. Spot D11 (Rv0815c) was identified as Putative thiosulfate sulfurtransferase. Spots D14 and D15 were Probable peptidyl-prolyl cis-trans isomerase A (Rv0009). Spots D16 and D17 (Rv1311) were identified as ATP synthase epsilon chain. Spots D18, D20 (Rv2031c) and D19 (Rv0251) were identified as 14 kDa antigen, heat shock protein HSPX. Spot D21 (Rv3418c) was 10 kDa chaperonin. Table 2 shows details of differentially expressed proteins identified by mass spectrometry.

**Table 2:** Details of over-expressed proteins of *M.tuberculosis *drug sensitive isolate identified by mass spectrometry

**Spot No**.	Protein identified	MASCOT Score	Nominal Mass (Da)	pI	Sequence Coverage %	Accession Number
D1	Glucose-6-phosphate isomerase (GPI)	136	59937	5.40	29%	Rv0946c

D2	Adenosylhomocysteinase (AdoHcyase)	87	54461	5.07	19%	Rv 3248c

D3	Adenosylhomocysteinase (AdoHcyase)	177	54461	5.07	38%	Rv 3248c

D4	Adenosylhomocysteinase (AdoHcyase)	188	54461	5.07	41%	Rv 3248c

D5	Adenosylhomocysteinase (AdoHcyase)	58	54461	5.07	13%	Rv 3248c

D6	Adenosylhomocysteinase (AdoHcyase)	60	54461	5.07	11%	Rv 3248c

D7	Hypothetical protein	20	40376	6.61	12%	Rv2896c

D8	Universal stress protein	113	31747	5.46	57%	Rv2623

D9	Antigen 85-C, Mycolyl Transferase 85c	85	36771	5.1	30%	Rv0129c

D10	Aspartate carbamoyltransferase (ATCase)	60	33798	6.60	24%	Rv1380

D11	Putative thiosulfate sulfurtransferase	37	31110	5.14	24%	Rv0815c

D12	Antigen 85-A FBPA (Mycolyl transferase 85A)	80	28634	5.3	25%	Rv3804c

D13	Hypothetical protein	21	26044	6.18	6%	Rv2558

D14	Probable peptidyl-prolyl cis-trans isomerase A	30	19285	5.80	20%	Rv0009

D15	Probable peptidyl-prolyl cis-trans isomerase A	110	19285	5.80	54%	Rv0009

D16	ATP synthase epsilon chain ATPC	51	13127	4.55	25%	Rv1311

D17	ATP synthase epsilon chain.	38	13127	4.55	26%	Rv1311

D18	Heat shock protein HSPX (alpha-crystallin homolog) (14 kda antigen) HSP16.3	108	16217	5.0	59%	Rv2031c

D19	14 kDa antigen (16 kDa antigen)	74	16217	5.00	38%	Rv0251c

D20	Heat shock protein HSPX (alpha-crystallin homolog) (14 kda antigen) HSP16. 3.	79	16217	5.0	37%	Rv2031c

D21	10 kDa chaperonin	67	10798	4.62	35%	Rv3418c

### Functional characterization of hypothetical proteins employing bionformatic tools

#### (a) BLAST Analysis

Prediction of probable function of hypothetical proteins was done using BLASTp search performed at NCBI server http://blast.ncbi.nlm.nih. The top five BLAST hits of all six mycobacterial proteins showed statistically significant alignment with 90-100% sequence coverage. Rv0036, Rv2032c, Rv2896c and Rv2558 though found to be highly conserved in mycobacterial species could not be assigned any function because the hits were identified as hypothetical proteins. BLAST hits of Rv0635 were found to be highly conserved in different mycobacterial species where it functions like 3R-hydroxyacyl-ACP dehydratase subunit HadA. BLAST hits of Rv1827 were highly conserved in mycobacterial sp. but attributed it diverse functional annotations like hypothetical protein sequences, Chain A Pknb-Phosphorylated protein, FHA (Forkhead-associated) domain containing protein. FHA domain is a phosphopeptide recognition domain found in many regulatory proteins. As BLAST analysis could not reveal probable function of many proteins, we also searched for the presence of motif in these proteins using MotifScan.

#### (b) MotifScan Analysis

MotifScan run was performed for all the hypothetical proteins, but no motif was found except in Rv0635. A motif YNNN_MF_00799 http://myhits.isb-sib.ch/cgibin/view_mot_entry?name=hamap:YNNN_MF_00799 was detected in Rv0635. This motif is conserved in UPF0336 family of proteins. The proteins of this family comprise *M. leprae, M. paratuberculosis, M. tuberculosis *and *M. bovis *in addition to many other species of bacteria. This motif is termed as MaoC_dehydratas domain which is a member of HotDog superfamily and is involved in oxidoreductase activity.

#### (c) Domain Analysis

We selected top five BLAST hits for probing into the conserved domains and hence annotating the function of each query protein http://www.ncbi.nlm.nih.gov/cdd. Rv0635 had a putative R_hydratase like conserved domain http://www.ncbi.nlm.nih.gov/Structure/cdd/cddsrv.cgi?uid=4803 which is found in the proteins belonging to HotDog superfamily. Analysis of conserved regions of BLAST hits of Rv1827 revealed the presence of FHA http://www.ncbi.nlm.nih.gov/Structure/cdd/cddsrv.cgi?uid=28942 and a phospopeptide binding site and analysis of Rv0036 revealed presence of two conserved domains, of MDMPI_N Superfamily and Radical_ SAM superfamily http://www.ncbi.nlm.nih.gov/Structure/cdd/cddsrv.cgi?uid=175426. In the top five BLAST hits of Rv2032c we found a conserved domain belonging to Nitro_FMN_reductase superfamily and a site which forms a dimer interface http://www.ncbi.nlm.nih.gov/Structure/cdd/cddsrv.cgi?uid=174256. A Lysine_decarbox conserved domain was found to be present in the top five BLAST hits of Rv2896c http://www.ncbi.nlm.nih.gov/Structure/cdd/wrpsb.cgi?RID=7FEA98PB016 while no conserved domains were found to be present in the top five BLAST hits of Rv2558.

## Discussion

In the present study we have analyzed the protein profiles of *M.tuberculosis *MDR and sensitive clinical isolates while infecting human macrophage-like THP-1 cells as a means of analyzing the environmental conditions faced by the pathogen during infection. This may contribute significantly in understanding whether mechanisms adopted by resistant and sensitive mycobacteria to survive and grow inside the host macrophages are similar or different and would help in finding new drug targets. The use of human THP-1 macrophage cell line, rather than human peripheral blood monocyte derived or alveolar macrophages, was necessary to provide sufficient cells to recover intracellular mycobacteria.

MALDI-MS identification of the intracellular MDR and sensitive isolates revealed that majority of the common proteins upregulated/expressed in the intracellular state belonged to intermediary metabolism and respiration category. Four proteins (adenosylhomocysteinase, aspartate carbomyltransferase, putatitive thiosulfate sulfurtransferase and universal stress protein) were present in both isolates and three of these belonged to intermediary metabolism and respiration category. Two proteins (alanine dehydrogenase and adenosine kinase) of intracellular MDR isolate and two (glucose-6-phosphate isomerase and ATP synthase epsilon chain) of intracellular sensitive isolate belonged to intermediary metabolism and respiration category. One protein (Peroxidase/Catalase) of intracellular MDR and three (HSPX, 14kDa antigen and 10kDa chaperonin) of sensitive isolate belonged to virulence, detoxification and adaptation category. ESAT-6 of intracellular MDR belonged to cell wall and cell processes category. Two proteins (Antigen 85-C and Antigen 85-A) of intracellular sensitive isolate were involved in lipid metabolism while probable peptidyl-prolyl cis-trans isomerase A was involved in information pathways. Four (Rv0635, Rv1827, Rv0036c and Rv2032) of intracellular MDR and two proteins (Rv2896c and Rv2558c) of sensitive isolate were hypothetical proteins which were functionally characterized using bioinformatic tools.

Adenosylhomocysteinase (SAHH) catalyzes the reversible hydrolysis of S-adenosylhomocysteine (SAH) into free adenosine (ADO) and L-homocysteine (HCY). SAH is produced from S-adenosylmethionine (SAM) as a by-product of SAM-dependent methyltransferase reactions. Methylation plays a role in a wide range of cellular processes, including DNA replication and repair, methionine metabolism, polyamine and phospholipid biosynthesis. SAHH is also considered "druggable" [14], has been shown to be essential for growth *in vitro *and appears to be upregulated in infected mouse lung tissue [15]. Aspartate carbamoyltransferase (also known as aspartate transcarbamoylase or ATCase) catalyzes the first step in the pyrimidine biosynthetic pathway. Thiosulfate sulfurtransferase is a Rhodanese-like protein which catalyzes transfer of sulfane sulfur from substrate to enzyme active site and then to a thiophilic acceptor. Much information on the functional role of rhodanese is not available however lack of rhodanase encoding gene has shown to affect the sensitivity of *Azotobacter vinelandii *to oxidative damages [16]. *M. tuberculosis *has universal stress proteins, whose function is unknown. Proteomic and transcriptomic analysis have shown that a number of these genes are significantly upregulated under hypoxic conditions and in response to nitric oxide and carbon monoxide, as well as during *M. tuberculosis *infection of macrophage cell lines, suggesting their probable role in persistence and/or intracellular survival [17].

NAD (H)-dependent L-alanine dehydrogenase catalyzes the oxidative deamination of L-alanine and reductive amination of pyruvate to generate alanine for protein and peptidoglycan synthesis and plays a key role in cell wall synthesis. Feng et al. [18] have shown its important role in alanine utilization and anaerobic growth in mycobacteria. Huttera and Dicka [19] using *M.smegmatis *reported its upregulated during oxygen depletion-induced dormancy. This indicates that during intracellular state bacilli are not metabolically inactive but maintain a low level metabolism to tide over the unfavorable condition. Adenosine kinase which catalyzes the phosphorylation of adenosine is important for the regulation of cellular levels of adenosine and its nucleotides and was found to be expressed by MDR isolate during intracellular state. Purine metabolism has been proposed as a drug target [20]. Glucose-6-phosphate isomerase (PGI) plays a central role in glycolysis and gluconeogenesis. Interruption of PGI gene resulted in glucose auxotrophy [21]. During mycobacterium infection in macrophages, a metabolic shift from a strict aerobic respiratory mode to anaerobic metabolism occurs, with a significant increase in the levels of glycolytic enzymes. During this stage, 70% of the organism's energy is derived from glycolysis. Thus, being central to the organism's survival, the enzymes involved in glycolysis are an attractive target for drug design. *M.tuberculosis *sensitive isolate showed an increased expression of ATP synthase epsilon chain. ATP synthase epsilon chain produces ATP in presence of a proton gradient across the membrane. Tran and Cook [22] showed that it is an essential gene in *M. smegmatis *during growth on nonfermentable and fermentable carbon sources. Gengenbacher et al. [23] showed that nutrient-starved, non-replicating *M.tuberculosis *requires respiration, ATP synthase and isocitrate lyase for maintenance of ATP homeostasis and viability.

Intracellular MDR isolate showed an increased expression of Catalase/peroxidase protein. Encoded by the *katG *gene it is an important virulence determinant *of M.tuberculosis *which protects against oxidative stress. Catalse/peroxidase activity has been shown necessary for growth and persistence in mice and guinea pigs [24] and in human peripheral blood monocyte [25]. Isoniazid, a widely used frontline antimycobacterial agent requires activation by catalase-peroxidase KatG before exerting a lethal effect. KatG couples the isonicotinic acyl with NADH to form isonicotinic acyl-NADH complex that ultimately confers antitubercular activity [26]. HSPX is a 14kDa antigenic stress protein induced by anoxia and has a proposed role in maintenance of long-term viability during latent, asymptomatic infections, in replication during initial infection of macrophages [12,27].

6 kDa early secreted antigenic targets (ESAT-6) which forms a heterodimeric complex with culture filtrate protein (CFP-10) was exclusively detected during intracellular growth of MDR isolate and is involved in host-pathogen interactions. They induce a strong T cell mediated response, involved in membrane/host-cell lysis and represent key virulence factors [28]. ELISA-IGRA test uses ESAT-6 and CFP-10 as stimulating antigens for detecting tuberculosis infection [29] and has the ability to discriminate between tuberculosis infection and previous use of BCG vaccine or atypical mycobacteria reactivity.

Antigen 85-C and Antigen 85-A mycolyl transferases constitute a major fraction of the secreted proteins of *M. tuberculosis *culture filtrate. Gene disruption studies encoding the three Ag85 components of *M. tuberculosis *suggests that Ag85A may be the most essential component for bacterial survival within macrophages. Ag85A and Ag85B have been reported to be ideal vaccine candidates in a number of studies [30,31]. Peptidyl-prolyl cis-trans isomerases catalyzes the interconversion of cis and trans peptide bonds and are therefore considered to be important for protein folding. They are also thought to participate in processes such as signalling, cell surface recognition, chaperoning and heat shock response [32]. 14 kDa antigen (16 kDa antigen) **is **believed to be involved in the initiation step of translation at high temperature and is possibly a molecular chaperone. Sharma et al. found that the expression of this protein was upregulated in drug -pressure of streptomycin [33]. Chaperonins form a sub-group of molecular chaperones and 10-kDa antigen has homology with the GroES or chaperonin-10 (Cpn 10) family of heat shock proteins [34]. 10-kDa antigen has been shown to be an important T-cell antigen in tuberculosis patients.

Further, intracellular resistant/sensitive *M.tuberculosis *hypothetical proteins were functionally characterized using bioinformatics tools. Rv0036, Rv2032c, Rv2896c and Rv2558 though found to be highly conserved in *Mycobacterium *species could not be assigned any function through BLASTp analysis. Motif Scan webserver also didn't find any motifs in these proteins while CDD found conserved domains in these except Rv2558. Top five BLAST hits of Rv0036 revealed the presence of two conserved domains, MDMPI_N and Radical_ SAM superfamily. No information is available on the function of proteins belonging to MDMPI_N Superfamily. As enzymes of Radical SAM superfamily catalyze steps in metabolism, DNA repair etc. [35], it can be inferred that Rv0036 may participate in such activities. Rv2032c showed a conserved domain belonging to Nitro_FMN_reductase superfamily. Proteins of this superfamily catalyze the reduction of flavin or nitrocompounds. Lysine_decarbox conserved domain was found in Rv2896c. This family includes proteins annotated as lysine decarboxylases, whose synthesis is enhanced on exposure to fluoroquinolones [36]. Rv0635 was found highly conserved in different *Mycobacterium *species where it functions like 3R-hydroxyacyl-ACP dehydratase subunit HadA. MotifScan analysis predicted the presence of YNNN_MF_00799 motif which is conserved in UPF0336 family of proteins. This motif is termed as MaoC_dehydratase domain which is a member of HotDog superfamily and is involved in oxidoreductase activity. CDD investigation also revealed the presence of R_hydratase like conserved domain in Rv0635. Dhillon and Bateman [37] unified numerous prokaryotic, archaeal and eukaryotic proteins and found that hotdog domain containing proteins wrap up a superfamily of thioesterases and dehydratases. Combining the results of BLASTp, MotifScan and CDD it can be inferred that Rv0635 codes for a metabolic enzyme which may be involved in dehydratase activities. BLAST hits of Rv1827 found it to be highly conserved in *Mycobacterium *species but attributed diverse functional annotations to it like hypothetical protein sequences, Chain A Pknb-Phosphorylated protein, FHA (Forkhead-associated) domain containing protein. MotifScan reported no motifs in Rv1827 while CDD analysis revealed the presence of FHA domain. It is a putative nuclear signalling domain and is known to mediate phosphorylation-dependent protein-protein interactions. Diverse functional attributes associated with FHA-domain containing proteins like cell shape regulation, signal transduction, ethambutol resistance etc. [38] and the expression of Rv1827 by MDR isolate during intraphagosomal state highlights the importance of this protein in cellular physiology of bacteria and its intracellular survival. Based on the bioinformatics findings we can predict that proteins encoded by Rv0036, Rv2032c, Rv0635, Rv1827 and Rv2896c genes are involved in cellular metabolism and help in intracellular survival of drug resistant/sensitive *M.tuberculosis*.

## Conclusions

Taken together, the identified proteins unique to intraphagosomal *M.tuberculosis *are likely to contribute to the adaptation of the bacteria to the milieu within phagosomes. The deduced functions of identified proteins are consistent with the conditions encountered within the macrophages. The induction of their expression observed *in vitro *may therefore represent a strategy employed by *M.tuberculosis *to promote the infection and to enhance intracellular survival. Identification of the exact role of these proteins will allow direct interference with their function to control intracellular growth of mycobacteria.

Mass spectrometry and bioinformatic characterization of both drug resistant and sensitive isolates of *M.tuberculosis *during intraphagosomal growth showed that majority of commonly expressed/upregulated proteins belonged to the cellular metabolism and respiration category. Though both the clinical isolates were from different clades, some proteins common in them were found overexpressed. It represents that some common mechanisms is adopted by sensitive/resistant mycobacteria for their survival within macrophages and thus could serve as important drug targets. The substantive response of the host immune system that includes both oxidative and nitrosative stress provokes *M.tuberculosis *to adopt metabolic enzymes to catabolize these toxic compounds and undergo fundamental physiological and metabolic changes to facilitate intracellular survival. Matteelli et al. [39] reported that TMC207 is a first-in-class anti-TB diarylquinoline with activity against drug-sensitive and resistant TB and appears to be safe and well tolerated. It inhibits the proton pump of mycobacterial ATP synthase, a critical enzyme in the synthesis of ATP for *M. tuberculosis*. Though knowledge is meagre regarding the metabolism *M. tuberculosis *during its residence in host cells but studies have shown that *M. tuberculosis *faces a hypoxic, carbon-poor, oxidative, and nitrosative environment in the host cells and organs. A close intersection exists between core intermediary metabolism and resistance to host-imposed biochemical stress in *M. tuberculosis *which has suitably adapted its metabolism for persistence in the human macrophage. Inhibitors of the metabolic enzymes therefore warrant exploration as suitable drug targets against drug-resistant and sensitive subpopulations of *M.tuberculosis*.

## Methods

### Source of culture

*M.tuberculosis *drug sensitive (to five first line drugs) (ST11/EAI3_IND family) and MDR (resistant to rifampicin, isoniazid and streptomycin) (ST288/CAS2 family) clinical isolates were procured from Mycobacterial Repository Center at National JALMA Institute for Leprosy and other Mycobacterial Diseases, Agra. Susceptibility testing was performed by conventional LJ proportion method [40]. Cultures were grown in Sauton's liquid medium at 37°C and harvested in late exponential phase (3 weeks) of growth [41,42]. These cultures were used for macrophage infection.

### Culture of cell line and infection with mycobacteria

The human leukemic macrophage like cell line THP-1 (kind gift from National Centre for Cell Science, Pune, India) was routinely maintained as suspended cells in RPMI 1640 media (Sigma, USA), supplemented with 10% v/v heat-inactivated foetal calf serum, 2 mM glutamine and 100 μg/ml antibiotic-antimycotic cocktail from Sigma (penicillin, streptomycin, amphotericin B) at 37°C in a CO_2 _humidified incubator. Cells were grown to a density of 2-5 × 10^6 ^cells/ml in 25 cm^2 ^flat-bottom tissue culture flasks and passaged every third day. Prior to infection with *M.tuberculosis *isolates the THP-1 cells were passaged at least three times in supplemented antibiotic-free RPMI 1640 growth medium before expansion into 75 cm^2 ^flat bottom tissue culture flasks containing 30 ml RPMI 1640 growth medium. The cells grown to a density of 2-5 × 10^6^/ml, were stimulated with 20 nM (12 ng/ml) phorbol 12-myristate13 acetate (PMA; Sigma-Aldrich, USA) for 24 hours so as to allow the cells to adhere [12]. Non-adherent cells were removed by washing twice in warm RPMI 1640 (at 37°C) and the resulting monolayers (approx.3-5 × 10^7^cells per flask) were covered with 30 ml supplemented RPMI 1640 growth medium. Mid-exponential phase mycobacterial cells (5 × 10^8 ^bacilli) recovered from Sauton's liquid medium by centrifugation at 5000 g for 10 minutes were resuspended in 1 ml RPMI 1640 growth medium and added to the adhered macrophage monolayer at an infection ratio of 10 bacilli per macrophage [43] and left to phagocytose for 12 hours (to ensure that sufficient mycobacteria are taken up by macrophages) at 37°C in a 5% humidified CO_2 _incubator. After 12 hours extracellular mycobacteria were removed by decanting the supernatant and extensively washing the adhered cells twice in warm RPMI 1640. The infected macrophages were replenished with 30 ml complete RPMI 1640 medium containing gentamycin (10 μg/ml) to prevent extracellular replication of mycobacteria. Macrophage viability (never fell below 80%) was assessed by trypan blue exclusion. Incubations were carried out for further 5 days at 37°C in a humidified CO_2 _incubator. After 5 days incubation, the supernatant was discarded and macrophages were washed and scrapped off in chilled Phosphate buffered saline (PBS) solution. The macrophages were then lysed by incubating for 5 minutes at 37°C in 0.05% SDS/PBS (w/v). Lysed cell suspension was transferred to 15 ml centrifuge tubes and centrifuged at 5000 g for 20 minutes. To remove any contaminating macrophage proteins, and ensure the recovery of intracellular mycobacteria only, pelleted mycobacteria were pooled and washed further two times in PBS containing 0.1% Tween 80 (w/v) and 0.1% (w/v) SDS. The washed mycobacterial pellet containing only the intracellular mycobacteria was finally collected.

### Mycobacterial cell lysate proteins

Cell lysate proteins were prepared according to the recommended protocol [44] with slight modification. Intracellular mycobacterial pellet was suspended (0.2 g/ml) in lysis buffer (50 mM Tris/HCl, pH 7.4 with 10 mM MgCl_2_, 1 mM PMSF and 1 mM EGTA) and sonicated for 15 minutes intermittently. Lysates were clarified by centrifugation at 10, 000 g for 30 minutes. Using similar protocol cell lysate proteins of broth- grown mycobacterial cells was also prepared. Liquid cultures of MDR and sensitive isolates grown in Sauton's liquid medium were harvested in mid exponential phase of growth. Cells were collected, washed in PBS and lysate proteins prepared.

### Protein precipitation with SDS-TCA-acetone

Cell lysate was treated with 1% SDS and then subjected to trichloro acetic acid (TCA)-acetone precipitation procedure [45]. 10% TCA was added to the cell lysate, the mixture was incubated at -20°C overnight and then precipitated protein was collected by centrifugation at 18, 000 g at 4°C for 15 minutes. It was again washed twice with 100% ice cold acetone and allowed to air dry. The protein pellet was suspended in appropriate volume of two-dimensional rehydration buffer (BIO-RAD, Hercules, CA, USA). Protein concentration was estimated using the Bradford assay [46].

### Two-dimensional gel electrophoresis (2-DE)

Isoelectric focusing (IEF) was carried out using the method of 'in gel rehydration' [47] with slight modifications. Immobilized pH gradient (IPG) strips of pH 4-7 and length 17 cm (BIO-RAD, Hercules, CA, USA) were rehydrated overnight at 20°C with 500 μg protein mixed with rehydration buffer. Strips were then focused on an IEF unit PROTEAN IEF Cell (BIO-RAD, Hercules, CA, USA) using the following four step programme: a) 0-250 V for 2 hours in linear mode; b) 250 V constant for 2 hours in rapid mode; c) 250-5000 V for 4 hours in linear mode; and d) 5000 V constant until 35 kVh reached. The current limit was set at 50 μA per strip. After IEF, IPG strips were equilibrated for 15 minutes in equilibration buffer I (6 M urea, 2% SDS, 0.375 M Tris; pH 8.8, 20% glycerol) containing 130 mM dithiothreitol (DTT) followed by equilibration buffer II containing 135 mM iodoacetamide instead of DTT for 15 minutes. Proteins were separated in second dimension on 12% SDS-PAGE [48] in a vertical electrophoretic dual gel unit PROTEAN II XI (BIO-RAD, Hercules, CA, USA) at constant voltage of 250 V for 5-6 hours. Gels were stained with Coomassie Brilliant Blue R250 to visualize proteins. Images of gels were acquired by Chemidoc (BIO-RAD) using Quantity One software (BIO-RAD, Hercules, CA, USA). 2 D gels were analysed using PDQuest Advanced software (BIO-RAD, Hercules, CA, USA). Gel images were also manually checked for artifactual spots, merged spots, and missed spots. Equal amount of protein was loaded in all gels and experiments were repeated at least three times.

### In-gel digestion with trypsin

Method of Shevchenko et al. [49] was followed with slight modifications. Protein spots of interest were excised from gels using spot picker 'Investigator ProPic' (Genomic Solutions, Huntingdon, UK) and collected in 96 well PCR plate. Digestion of proteins and spotting of peptides on MALDI-TOF target plate was carried out using protein digester 'Investigator ProPrep' (Genomic Solutions, Huntingdon, UK). The gel plugs were destained and dehydrated by washing three times (~10 minutes) with 25 mM NH_4_HCO_3_-50% acetonitrile (ACN) (1:1). Dried gel plugs were treated with freshly prepared 10 mM DTT in 50 mM NH_4_HCO_3 _for 45 minutes at 56°C. After incubation, the DTT was replaced quickly by the same volume of freshly prepared 55 mM iodoacetamide in 50 mM NH_4_HCO_3 _for 30 minutes and then dehydrated with 100% ACN. The dried gel pieces were incubated for 12 hours at 37°C with 25 mM NH_4_HCO_3 _containing 0.02 μg/μl of mass spectrometry grade trypsin (Promega, Madison, WI, USA). The resulting peptides were extracted twice from the gel pieces, using peptide extraction buffer [1:1 mixture of 70% ACN and 0.1% trifluoroaceticacid (TFA)].

### Mass spectrometry

Digested samples were desalted and concentrated on C-18 ZipTips (Millipore, Billerica, MA, USA) using the manufacturer's protocol. ZipTips were eluted on MTP 384 target plate with 2 μl of a-cyano-4-hydroxycinnamicacid (HCCA) (Sigma-Aldrich, USA) saturated solution dissolved in 50% ACN and 0.2% TFA. Mass spectra of digested proteins were acquired using Autoflex II TOF/TOF 50 (Bruker Daltonik GmbH, Leipzig, Germany) in positive reflectron mode, in the detection range of 500-3000 m/z. External calibration to a spectrum, acquired for a mixture of peptides with masses ranging from 1046 to 2465 Da, was done prior to acquisition. The proteolytic masses obtained were then processed through Flex Analysis v.2.4 programme for peak detection of proteins. Peak detection in MALDI spectra and submission of peak lists to the Peptide mass fingerprint (PMF) Mascot server were done using the Mascot Wizard program (Matrix Science, U.K). Peptide mass tolerance was set to 50 ppm with carbamidomethyl-cystein set as fixed modification, oxidation of methionine as variable modification and 1 missed cleavage site was allowed.

### Computational analysis of hypothetical proteins employing bioinformatics tools

Computational analysis of the differentially expressed hypothetical proteins was carried out using softwares and web-servers like BLASTp, MotifScan and Conserved Domain Database (CDD). Protein sequences of all hypothetical proteins were retrieved from Tuberculist server http://genolist.pasteur.fr/TubercuList/ hosted by Pasteur Institute, Paris for whole annotated genome of H37Rv.

#### (a) BLAST Analysis

The prediction of probable function of hypothetical proteins was done using BLASTp search [50] performed at NCBI server http://blast.ncbi.nlm.nih. Protein sequence of hypothetical proteins was retrieved from Tuberculist server http://genolist.pasteur.fr/TubercuList. Top five BLAST hits were selected for annotating the function. BLAST runs were performed at NCBI server using the default threshold E-value of 10 and inclusion of threshold value of 0.005.

#### (b) Motif Scan Analysis

The motif search was done using HAMAP profiles and PROSITE patterns motif databases [51].

#### (c) Domain Analysis

The Conserved Domain Database (CDD) of NCBI which is a database of multiple sequence alignment [52] was used to predict functions of hypothetical proteins. Top five BLAST hits were selected for probing into the conserved domains and functional annotation http://www.ncbi.nlm.nih.gov/cdd.

## Competing interests

The authors declare that they have no competing interests.

## Authors' contributions

NS carried out the experiments, participated in the data analysis and drafted the manuscript. PS helped in mass spectrometric experiments and MK in bioinformatic analysis. BJ helped in carrying out cell line experiments and critical review of the manuscript. DB conceived and designed the study, interpreted the experiment data and drafted the manuscript. All authors read and approved the final manuscript.
